# Supplementary material for: Study of the Female Sex Survival Advantage in Melanoma—A Focus on X-Linked Epigenetic Regulators and Immune Responses in Two Cohorts
Source: Cancers (Basel). 2020 Jul 28;12(8):2082. doi: 10.3390/cancers12082082 (PMC7464825; doi:10.3390/cancers12082082)

Supplementary Figures and Tables:


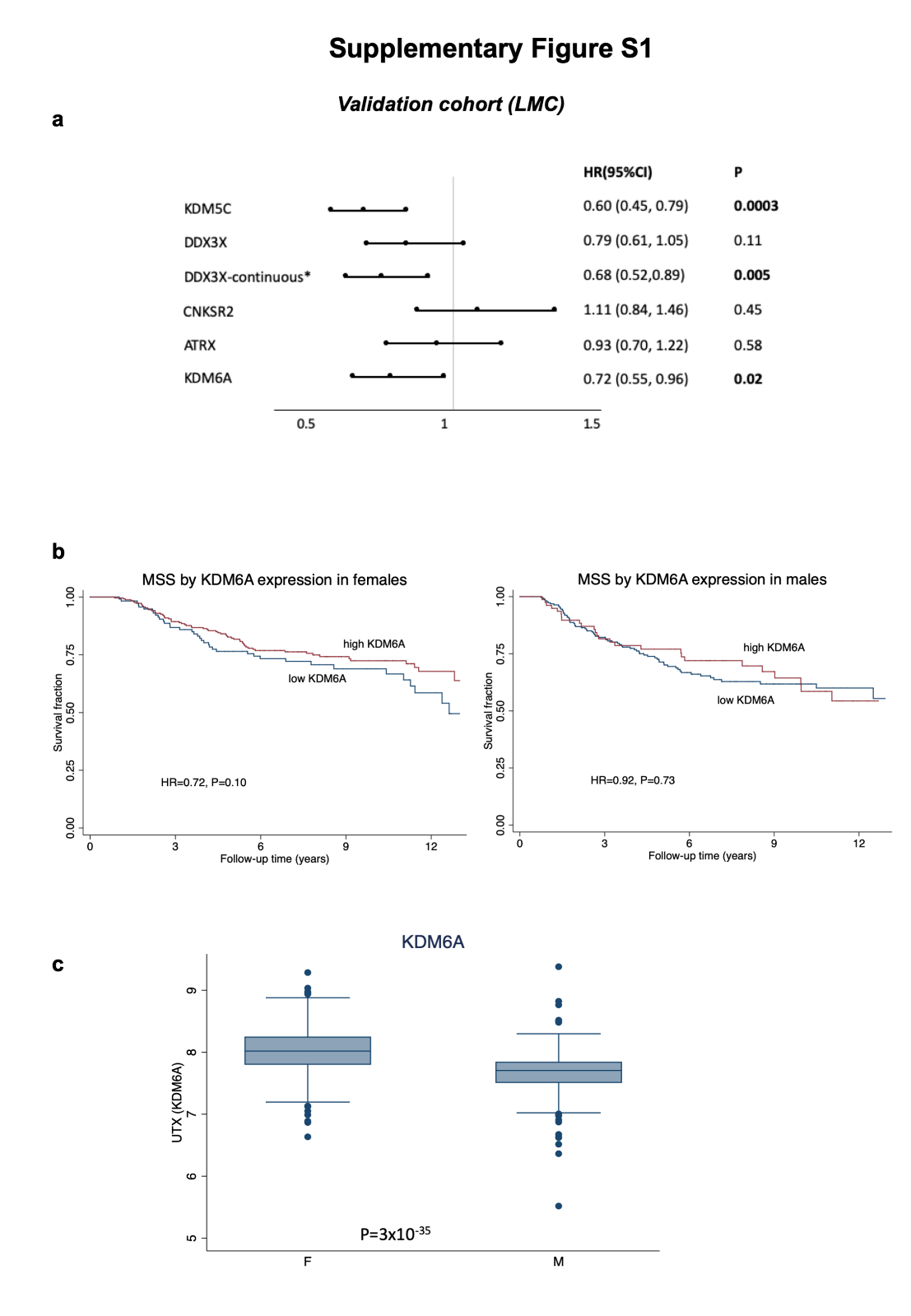


**Supplementary Figure S1:** Analysis of survival of the selected X-linked genes in LMC cohort**. a**) Melanoma specific survival (MSS) is shown as a forest plot representing the hazard ration of the indicated genes. Median split used to dichotomize and *P* < 0.05 refers to significance. **b**) MSS by KDM6A expression in females and males primary tumours. **c**) *KDM6A* mRNA expression based on sex. Unpaired t-test refers to the significance.


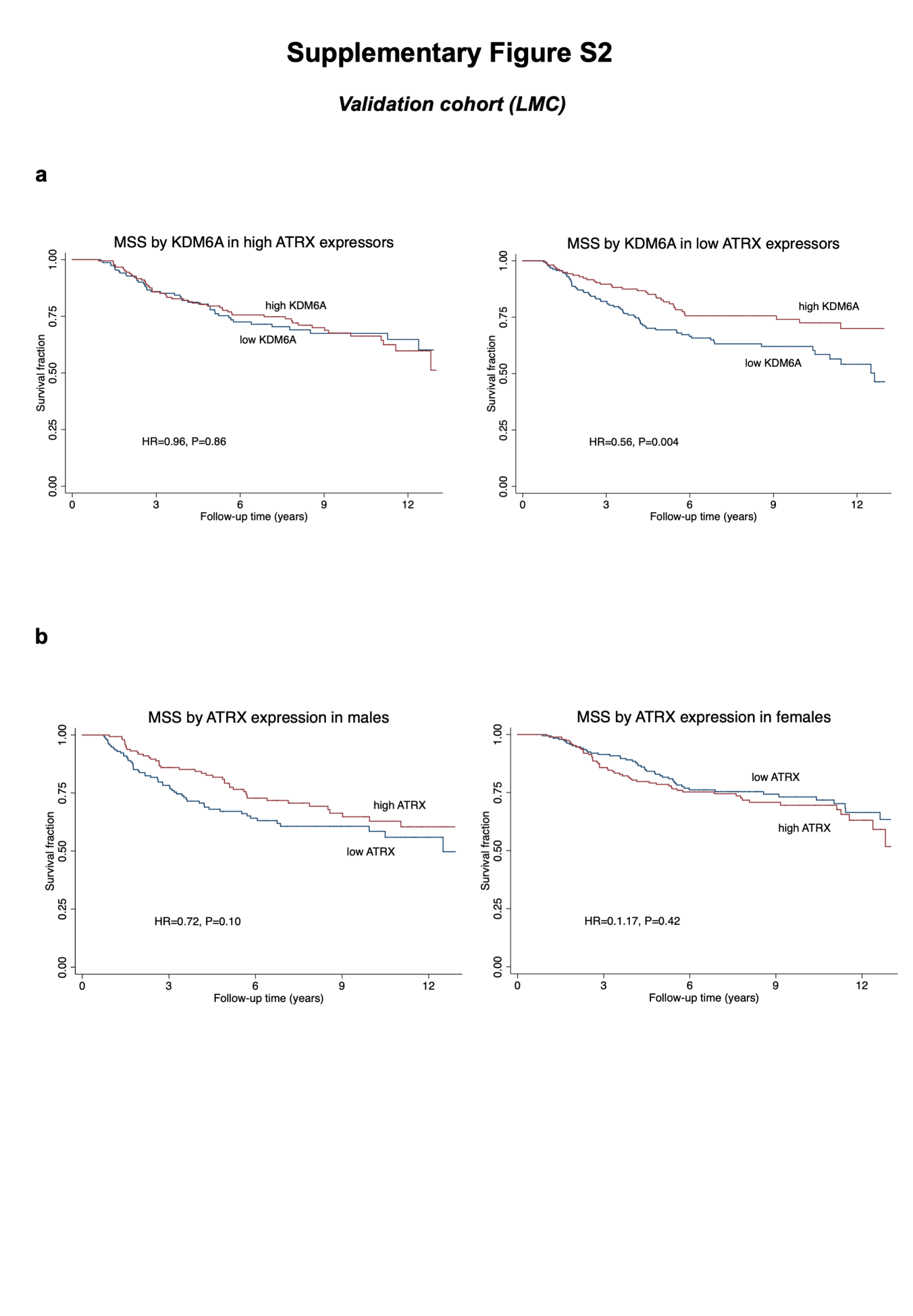


**Supplementary Figure S2:** KDM6A expression improves patient survival only when ATRX is low. **a**) KM plots representing MSS by *KDM6A* and *ATRX* expression **b**) by *ATRX* and sex. *P* < 0.05 refers to significant statistical interaction on MSS.


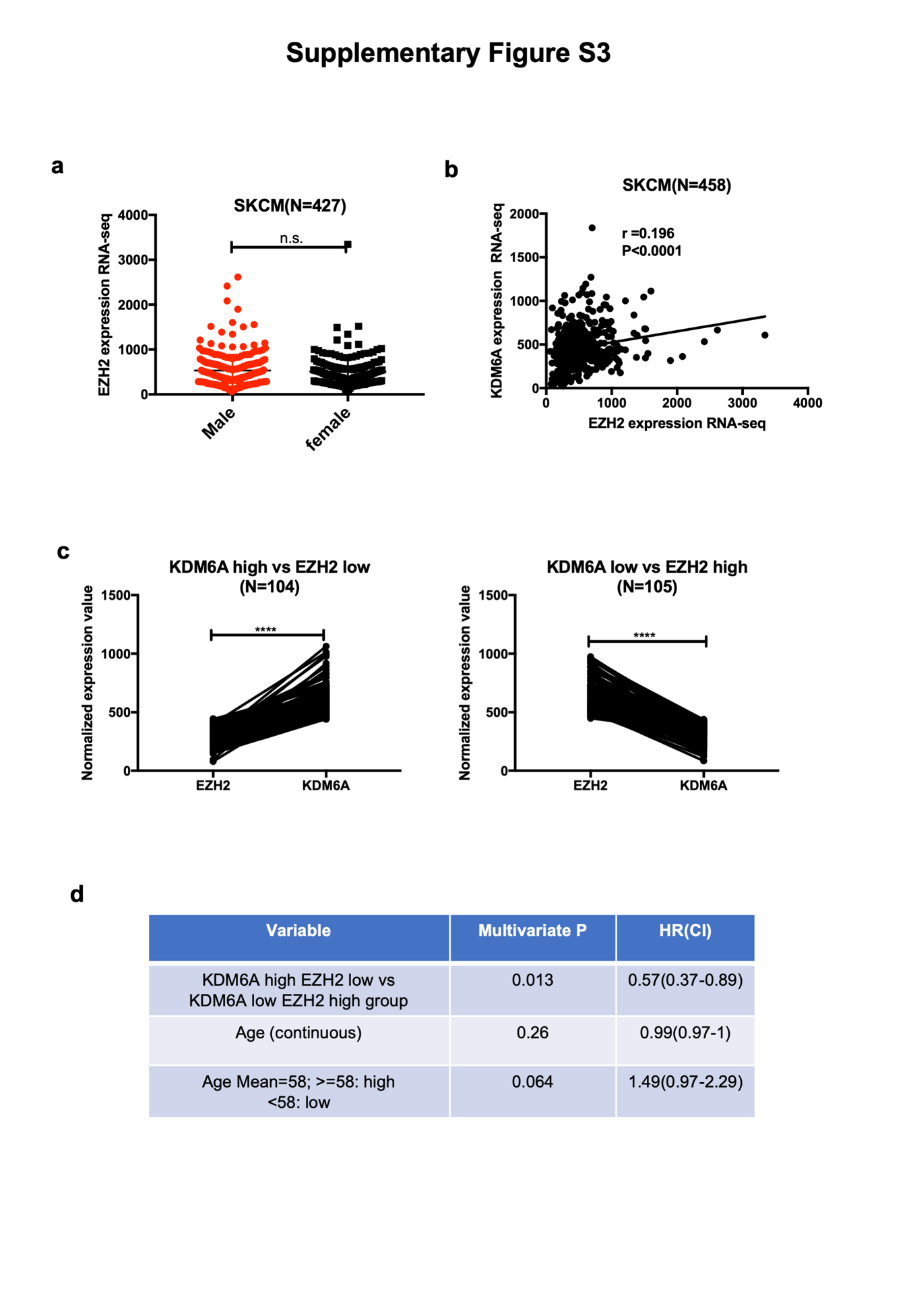


**Supplementary Figure S3:** Differential expression of *EZH2* and correlation with *KDM6A* and age. **a**) A dot plot showing differential expression of *EZH2* based on sex. **b**) Correlation *EZH2* with *KDM6A* in the SKCM cohort. **c**) Paired expression analysis of KDM6A high vs EZH2 low group and KDM6A low vs EZH2 high group. A paired t-test was performed to compute statistical significance and *****p* < 0.0001. d) Multivariate cox regression analysis of KDM6A high-EZH2 low group vs KDM6A low-EZH2 high group with age as either continuous or >= mean <= mean variable. HR = Hazard ratio; CI = Confidence Interval. *P* < 0.05 refers to significance.


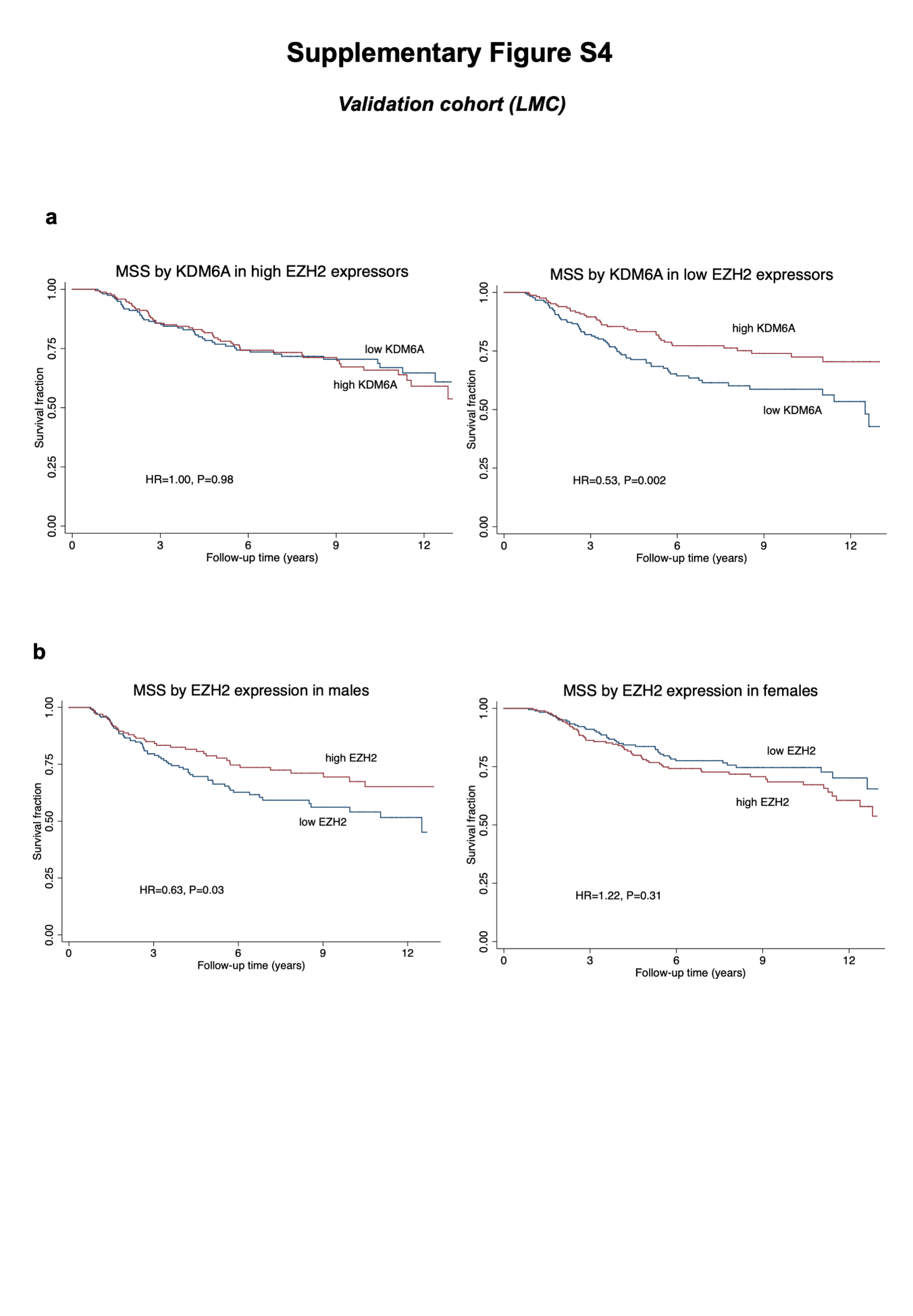


**Supplementary Figure S4:** KDM6A expression improves patient survival only when EZH2 is low **a**) KM plots showing MSS based on either *KDM6A* and *EZH2* or **b**) sex and *EZH2* interaction. *P* < 0.05 refers to significant interaction between two variables on MSS.


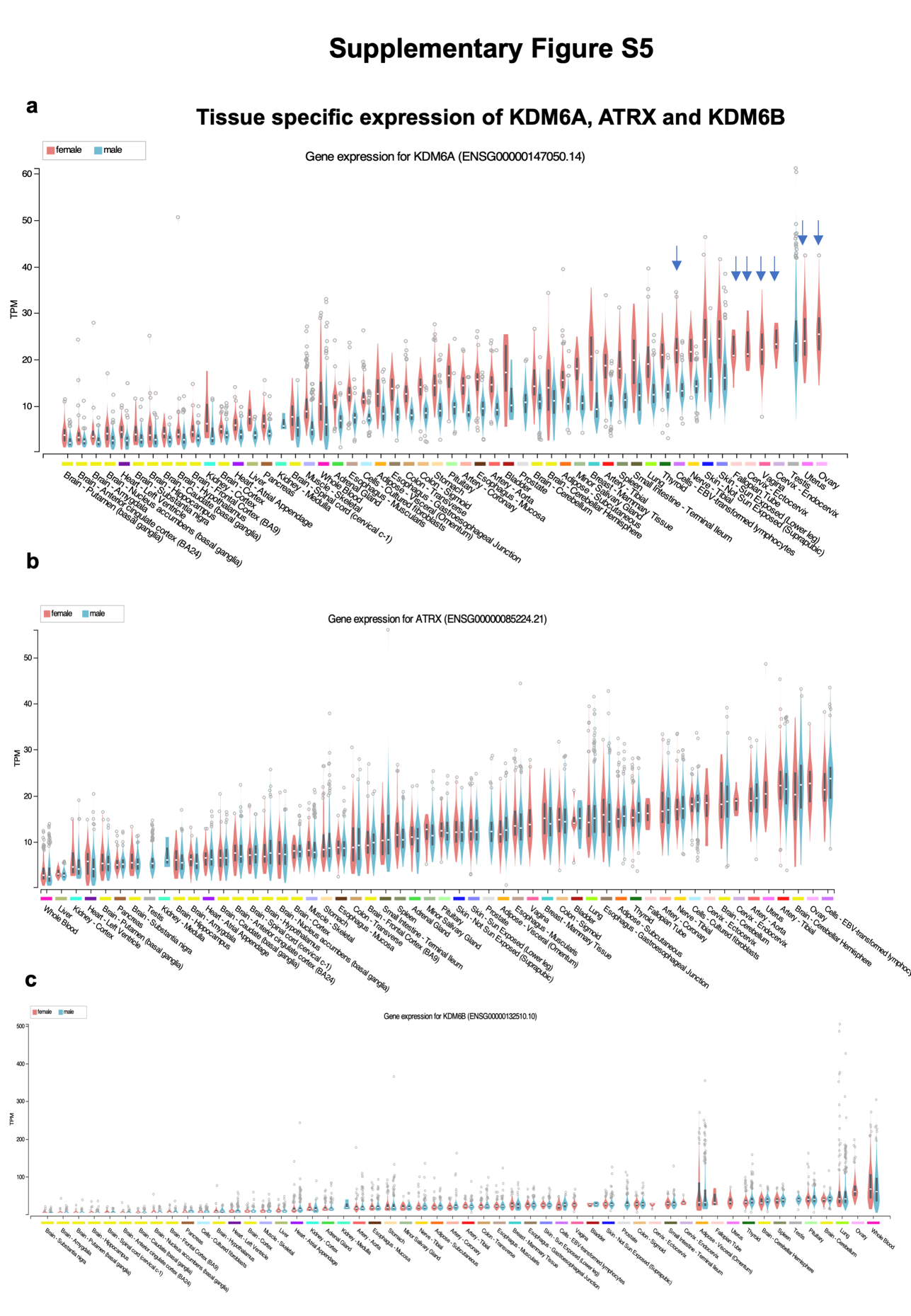


**Supplementary Figure S5**: **a**) *KDM6A* **b**) *ATRX* and **c**) *KDM6B* gene expression in different human tissues. The data was retrieved from GTEx portal [29] . Gene expression was sorted based on sex. TPM, transcript per million.

**Supplementary Table S1:** Overall survival analysis of KDM6A, ATRX and EZH2 in the SKCM primary and metastases cohort. Logrank *P* < 0.05 refers to significance.


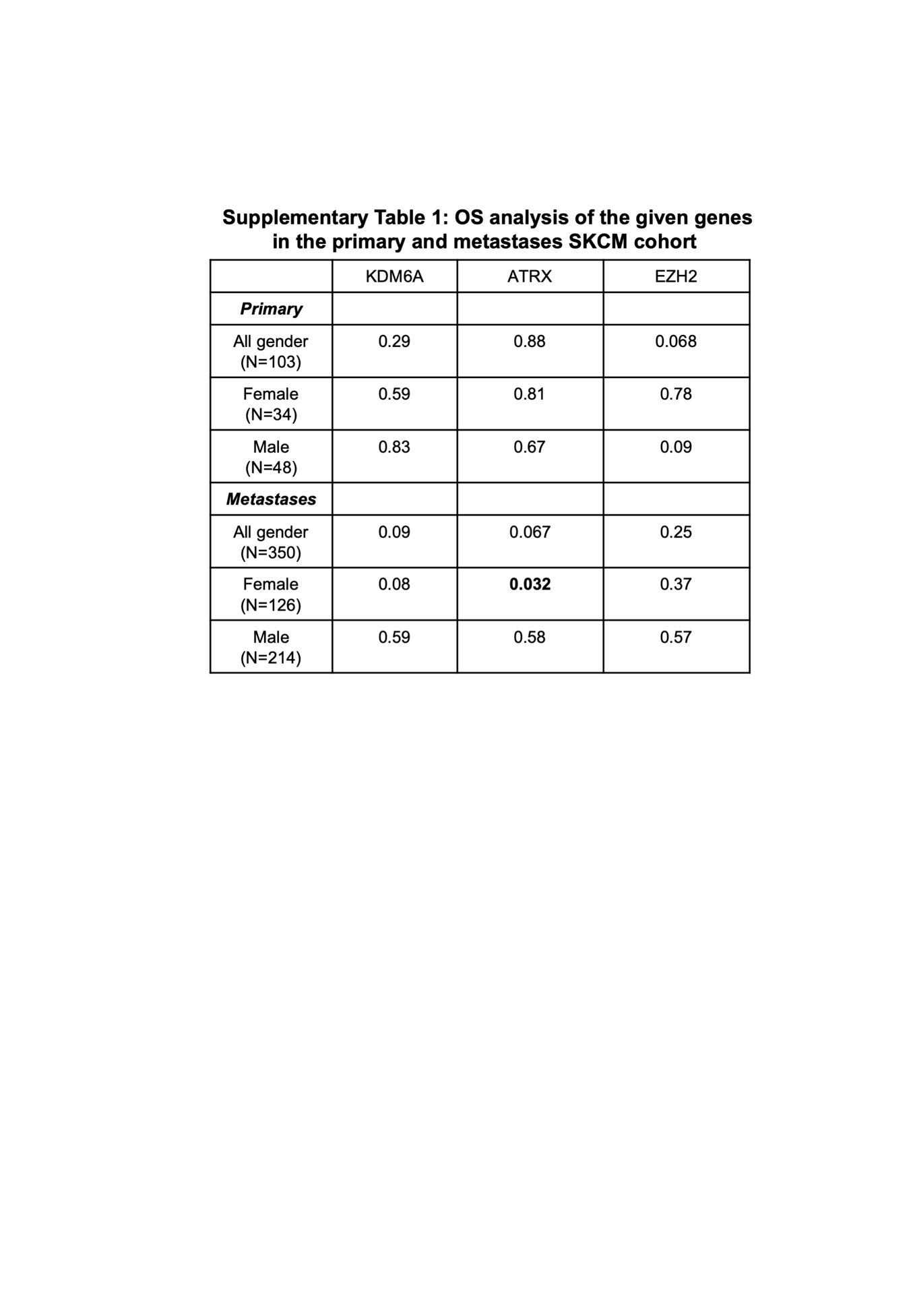


**Supplementary Table S2:** Correlation of the selected genes with **a**) immune cell subset and **b**) immune cell markers in the LMC cohort


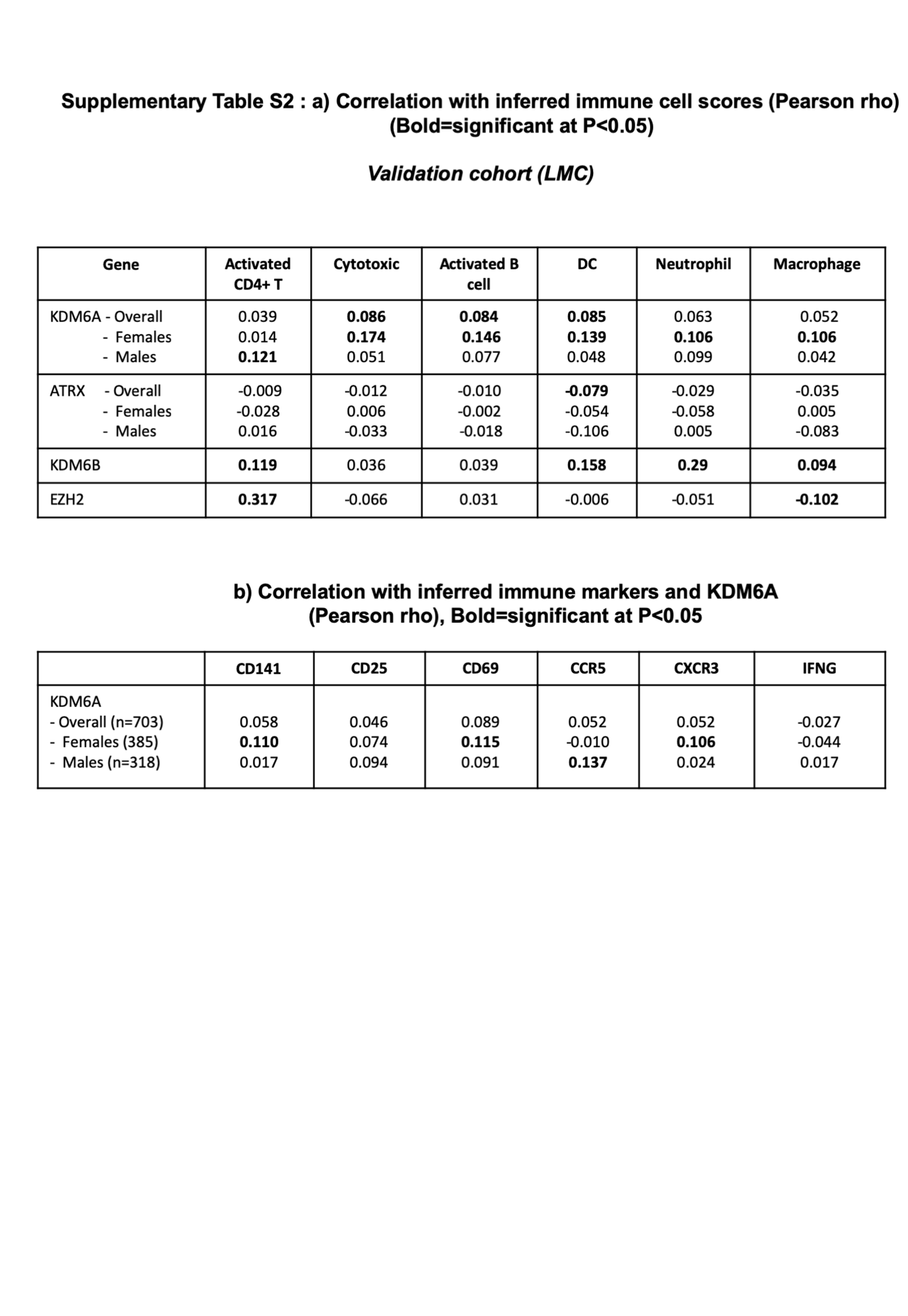


**Supplementary Table S3:** Clinical characterization of the SKCM and LMC cohort.


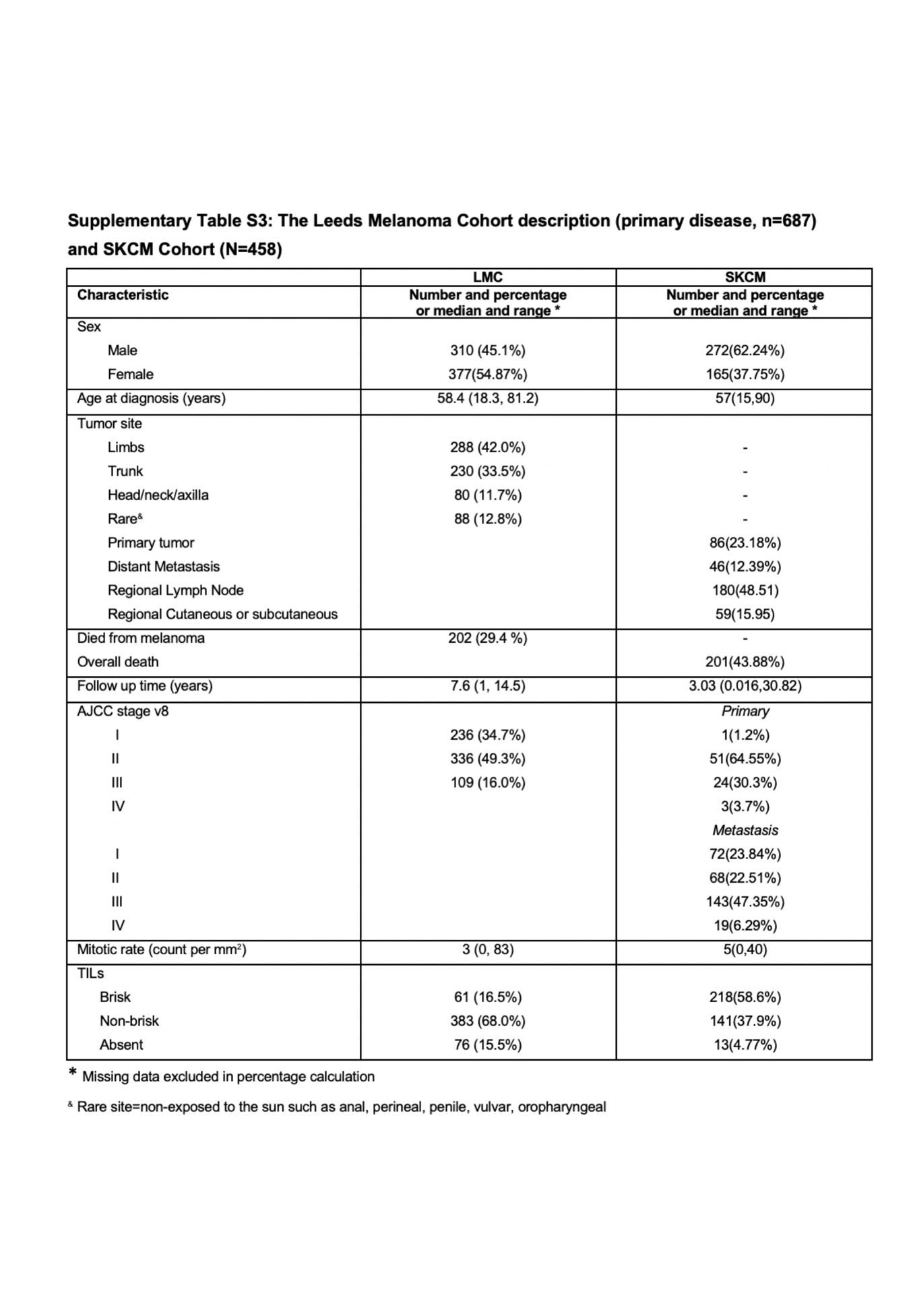

Supplement: Supplementary file 1 [file cancers-12-02082-s001.zip › Supplementary/Emran et al_Supplementary_Cancers.docx]
